# Relationship between sense of coherence and health-related behaviours in adolescents and young adults: a systematic review

**DOI:** 10.1186/s12889-022-12816-7

**Published:** 2022-03-10

**Authors:** Henrique da-Silva-Domingues, Rafael del-Pino-Casado, Pedro Ángel Palomino-Moral, Catalina López Martínez, Sara Moreno-Cámara, Antonio Frías-Osuna

**Affiliations:** Department of Nursing, University of Jaén, Jaén, Spain

**Keywords:** Sense of coherence, Health promotion, Adolescent health, Young adults, Risk behaviors, Literature review

## Abstract

**Background:**

The sense of coherence is developed through the learning process and contributes to the positioning of individuals in the health-disease continuum, facilitating successful and adaptive personal outcomes. Health-related behaviours represent a health determinant of utmost importance for public health and the development of adolescent and youth health promotion policies, as they are related to the main risk factors and problems of morbidity and mortality in our society. Previous studies have analysed the relationship between sense of coherence and only some individual health outcomes such as oral health, the relationship of sense of coherence with smoking and alcohol consumption, concluding that salutogenic factors are related to quality of life and preventive behaviours. The aim of this systematic review was to describe the relationship of sense of coherence with different health-related behaviours investigated so far in the adolescent and youth population.

**Methods:**

A systematic review was carried out in databases (PubMed, CINAHL, Scopus and PsycInfo) and in the bibliographies of the retrieved articles, without limitation of time or language. Associations between sense of coherence and health-related behaviours have been assessed.

**Results:**

A total of 1214 investigations were reviewed and 21 of them were included in this systematic review. The relationship between sense of coherence and eight health-related behaviours were identified (alcohol use, physical activity, tobacco use, eating habits, rest periods, use of illegal substances, behaviours related to oral health and time spent in games on the computer).

**Conclusions:**

Our results increase the available evidence and support the solid relationship of the sense of coherence with health behaviours both as a protective factor against risk behaviours and for its positive association with preventive and health promoting behaviours of adolescents, young adults and university students.

## Background

The Salutogenic model was proposed by Aaron Antonovsky [1] to explain the mechanisms that lead to health and the processes of active adaptation of the person to the environment. The central construct is the Sense of Coherence (SOC), a global and attitudinal orientation of the person through which life is understood as more or less comprehensible, significance and manageable; the SOC includes the availability of personal resources to meet the adaptation demands, considered by the person as challenges worthy of investment and commitment [2, 3]. The original scale that measures SOC is the Orientation to Life Questionnaire [4], with 29 items (SOC-29) that has solid research on the theoretical construct and its psychometric properties [5, 6]; subsequently, the abbreviated version of 13 items (SOC-13) [4] was developed. Both instruments collect the three components of the SOC: comprehensibility, manageability and significance; the available research shows that they are reliable, valid and cross-culturally applicable instrument [4].

SOC is strongly related to perceived health and life quality [7, 8]; this relationship is manifested in the studied populations regardless of age, sex, ethnicity, nationality and the type of study design [9]. SOC is developed through the learning process and contributes to the positioning of people in the health-disease continuum, facilitating personal results of success and adaptation [4]. The transition of adolescence and youth occupy a place of special interest in research on the relationship between SOC and health [10], since it is at this stage that the process of SOC acquisition and the development of health-related behaviors takes place, a process that is influenced by the social determinants of health [11]. This process conditions the acquisition of a more or less strong SOC to cope with life stressors [10], which conditions the adoption of certain health behaviors that will have repercussions on future health and wellbeing [12–14]. Health-related behaviors represent a health determinant of utmost importance for Public Health and the development of adolescent and youth health promotion policies [15], as they are related to the main risk factors and morbidity and mortality problems in our society [16, 17]. Currently, youth (population between 15 and 24 years old) represents 16% of the world’s population and it is estimated that this percentage will reach 23% by the year 2030 [18].

Previous research has studied the association of SOC with various variables (demographic, family, school, classmates, neighborhood, life quality, mental health) [19, 20]. Previous reviews have analyzed the relationship between SOC and some health outcomes such as oral health [21–23], concluding that salutogenic factors are related to life quality and oral-dental preventive behaviors during childhood and adolescence.

Given the need to understand the relationship of SOC with health-related behaviors in the adolescent and young adults, it is necessary to investigate the studies on the subject developed to date; at present, only the work of Länsimies et al. [20] has reviewed the relationship between SOC and health behaviors, showing the relationship of SOC with smoking habits, alcohol consumption and oral health care. The results of this study cover participants between the ages of 13 and 18, and its review is limited between the years 2007 and 2014. For all these reasons, the present research aims to know and expand the available knowledge of the research that relates SOC with the different health behaviors investigated so far in the population of adolescents and young adults.

## Methods

A systematic review was carried out to determine the available knowledge on the relationship between SOC and health behaviors in adolescents and young adults based on original studies. In terms of age, we followed the guidelines of the World Health Organisation [24], which defines young people as those between 10 and 24 years of age. In addition, considering that youth is the stage of university education, we decided to include studies on the university population in our review.

A systematic review was carried out following the methodology proposed in the PRISMA statement [25]. The search included the bibliographic databases PubMed, CINAHL, Scopus and PsycInfo, without using filters on language or temporal delimitation, in addition, a reverse search was carried out in the bibliography of the articles retrieved. Table 1 shows the search strategies used.

**Table 1:** Search strategies used for the different databases

Database	Search string	Date of search	Recovered record
PUBMED	(Young Adult[MH] OR Young Adult*[TIAB] OR Young adults[TIAB] OR Younger[TIAB] OR Adolescent[MH] OR Adolescen*[TIAB] OR Teens[TIAB] OR Teen[TIAB] OR university student*[TIAB] OR Adolescent Health[MH]) AND (sense of coherence[MH] OR sense of Coherence[TIAB] OR Salutogenesis[TIAB] OR SOC[TIAB]) AND (Health Risk Behaviors[MH] OR Health Risk behavior*[TIAB] OR Health Risk behaviour*[TIAB] OR healthy Habit*[TIAB] OR Healthy lifestyle[MH] OR Healthy lifestyle[TIAB] OR Alcohol Drinking[MH] OR Alcohol Consumption[TIAB] OR Alcohol Drinking Habit*[TIAB] OR Alcoholic Beverages[MH] OR Alcoholic Beverage[TIAB] OR Smoke[MH] OR Tobacco Smoking[MH] OR Smoking, Tobacco[TIAB] OR Exercise[MH] OR Exercises[TIAB] OR Physical Activit*[TIAB] OR Diet, Healthy[MH] OR Healthy Diet[TIAB] OR Healthy Diets[TIAB] OR Healthy Nutrition[TIAB] OR (Relaxation[MH] OR Relaxations[TIAB] OR sleep[MH] OR Sleeping Habits[TIAB] OR Sleep Habits[TIAB]) OR Safety[MH] OR Safeties[TIAB] OR Substance-Related Disorders[MH] OR Drug Abuse[TIAB] OR Drug Dependence[TIAB] OR Drug Addiction[TIAB] OR Substance Abuse*[TIAB] OR Health Promotion[MH] OR Promotion of Health[MH] OR Health Promotions[TIAB])	31/07/2020	149
CINHAL	(MH Young Adult OR AB Young Adult OR AB Young adults OR AB Younger OR MH Adolescence OR AB Adolescence OR AB Teen* OR MH Students OR AB Students, College OR MH Students, Graduate OR MH Adolescent Health) AND (AB sense of coherence OR AB Salutogenesis OR AB coherence sense OR AB SOC) AND (AB Health Risk Behaviors OR MH Risk Taking Behavior OR MH Health behavior OR AB Health Behavior OR AB healthy Habit OR MH Life Style OR AB Life Style OR MH Alcohol Drinking OR MH Alcohol Drinking in College OR AB Alcohol Drinking in College OR AB Binge Drinking OR MH Alcoholic Beverages OR MH Smoke OR MH Smoking OR MH Tobacco OR MH Exercise OR AB Exercise OR AB Physical Activity OR AB Diet, Health OR AB Healthy Diet* OR MH Adolescent Nutrition OR MH Eating Behavior OR MH Relaxation OR AB Relaxation OR MH Sleep OR AB Sleep OR AB Sleeping Habits OR AB Sleep Habits OR MH Safety OR MH Safety Precautions OR AB Individual Safety OR MH Substance Use Disorders OR MH Substance Abuse OR MH Substance Dependence OR AB Drug Abuse OR MH Health Promotion OR AB Promotion of Health OR AB Health Promoting Behavior)	31/07/2020	154
PsycInfo	(AB(Young Adult) OR AB(Young adults) OR AB(Younger) OR AB(Adolescent) OR AB(Teens) OR AB(Teen) OR AB(university student) OR SU(Adolescent Health) OR SU(Adolescent Behavior*) OR AB(Behavior*, Adolescent)) AND (SU(sense of coherence) OR AB(sense of coherence) OR AB(Salutogenesis) OR AB(coherence sense)OR AB(SOC)) AND (SU(Health Behavior) OR AB(Health Behavior) OR SU(Adolescent Health) OR AB(Lifestyle Changes) OR AB(healthy Habit) OR AB(Healthy lifestyle) OR SU(Alcohol Drinking Attitudes) OR AB(Drinking Attitudes) OR AB(Drinking Behavior) OR AB(Alcohol Drinking Patterns) OR SU(Alcohol Drinking Patterns) OR SU(Alcoholic Beverages) OR AB(SMOKE) OR SU(Tobacco Smoking) OR AB(Tobacco Smoking) OR AB(Cigarette Smoking) OR AB(Tobacco Use Disorder) OR SU(Exercise) OR AB(Exercise) OR AB(Physical Exercise) OR AB(Diet*, Healthy) OR AB(Healthy Diet) OR AB(Healthy Nutrition) OR SU(Obesity) OR AB(Diets) OR SU(Nutrition) OR SU(Relaxation) OR AB(Relaxations) OR SU(sleep) OR SU(Sleep Wake Disorders) OR AB(Sleep Disorders) OR SU(Safety) OR AB(SAFETIES) OR SU(Substance-Related Disorders) OR AB(Substance Abuse and Addiction Measures) OR SU(Substance Use Disorder) OR AB(Drug Use Disorder) OR SU(Drug Abuse) OR AB(Substance Abuse) OR AB(Drugs) OR SU(Drug Addiction) OR SU(Health Promotion) OR AB(Health Promotion) OR AB(Health Behavior))	31/07/2020	344
SCOPUS	(INDEXTERMS(Young Adult) OR TITLE-ABS-KEY(Young Adult) OR TITLE-ABS-KEY(Young adults) OR TITLE-ABS-KEY(Younger) OR INDEXTERMS(Adolescent) OR TITLE-ABS-KEY(Adolescen*) OR TITLE-ABS-KEY(Teens) OR TITLE-ABS-KEY(Teen) OR TITLE-ABS-KEY(university student*) OR INDEXTERMS(Adolescent Health)) AND (INDEXTERMS(sense of coherence) OR TITLE-ABS-KEY(sense of Coherence) OR TITLE-ABS-KEY(Salutogenesis) OR TITLE-ABS-KEY(coherence sense) OR TITLE-ABS-KEY(SOC)) AND (INDEXTERMS(Health Risk Behaviors) OR TITLE-ABS-KEY(Health Risk behavior*) OR TITLE-ABS-KEY(Health Risk behaviour*) OR TITLE-ABS-KEY(healthy Habit*) OR INDEXTERMS(Healthy lifestyle) OR TITLE-ABS-KEY(Healthy lifestyle) OR INDEXTERMS(Alcohol Drinking) OR TITLE-ABS-KEY(Alcohol Consumption) OR TITLE-ABS-KEY(Alcohol Drinking Habit*) OR INDEXTERMS(Alcoholic Beverages) OR TITLE-ABS-KEY(Alcoholic Beverage) OR INDEXTERMS(Smoke) OR INDEXTERMS(Tobacco Smoking) OR TITLE-ABS-KEY(Smoking, Tobacco) OR INDEXTERMS(Exercise) OR TITLE-ABS-KEY(Exercises) OR TITLE-ABS-KEY(Physical Activit*) OR INDEXTERMS(Diet, Healthy) OR TITLE-ABS-KEY(Healthy Diet*) OR TITLE-ABS-KEY(Healthy Nutrition) OR INDEXTERMS(Relaxation) OR TITLE-ABS-KEY(Relaxations) OR INDEXTERMS(sleep) OR TITLE-ABS-KEY(Sleeping Habits)OR TITLE-ABS-KEY(Sleep Habits) OR INDEXTERMS(Safety) OR TITLE-ABS-KEY(Safeties) OR INDEXTERMS(Substance-Related Disorders) OR TITLE-ABS-KEY(Drug Abuse) OR TITLE-ABS-KEY(Drug Dependence) OR TITLE-ABS-KEY(Drug Addiction) OR TITLE-ABS-KEY(Substance Abuse*) OR INDEXTERMS(Health Promotion) OR INDEXTERMS(Promotion of Health) OR TITLE-ABS-KEY(Health Promotions))	31/07/2020	567

Inclusion criteria were defined as those original articles that investigated the SOC construct measured by any of its validated scales (SOC-13 and SOC-29), relating it to at least one health behavior and referring to the adolescent population, young people and university students.

Criteria based on Higgins’ indications [26] and the Cochrane Handbook of Systematic Reviews were used as listed below: [27] 1) The original study should have a representative sampling (probabilistic). 2) Explanation of the confidence and validity of the measures (content validity and internal consistency in the target population or similar). 3) That the study takes into account the control of confounding factors. For longitudinal studies, two additional criteria were also taken into account: 4a) Follow-up of at least 6 months and 4b) Follow-up rate of at least 80% of the original participating population. Thus, the studies were classified as high quality when they met all the evaluated criteria, medium quality when one of the defined criteria was not met and low quality when two or more quality criteria were not met. The review and selection of the studies were carried out independently by two investigators; disagreements were resolved by consensus.

Data were extracted from the studies and collected in an Excel data sheet previously prepared for this purpose. The variables collected were: authors’ names, year of publication, country and study design, independent variable (SOC), dependent variable studied (health-related behavior), sampling and data collection method, key findings and correlation coefficient. These data were collected independently by two investigators; in case of disagreement, joint analysis was performed until consensus was reached.

It was observed that the included studies differed from each other in terms of the data collection methods and the statistics used for the analysis of the studies. The heterogeneity of the measurements and the results of the studies did not allow a statistical method to be used to combine the data. Consequently, it was not possible to pool the investigations for a meta-analysis. Therefore, the data were pooled and synthesized narratively. In the first phase of the analysis, the articles were read in their entirety to obtain a complete picture of the results. In the second phase, the information was tabulated and then the dependent variables that had been related to SOC were listed.

## Results

### Characteristics of the selected studies

**Fig. 1:**
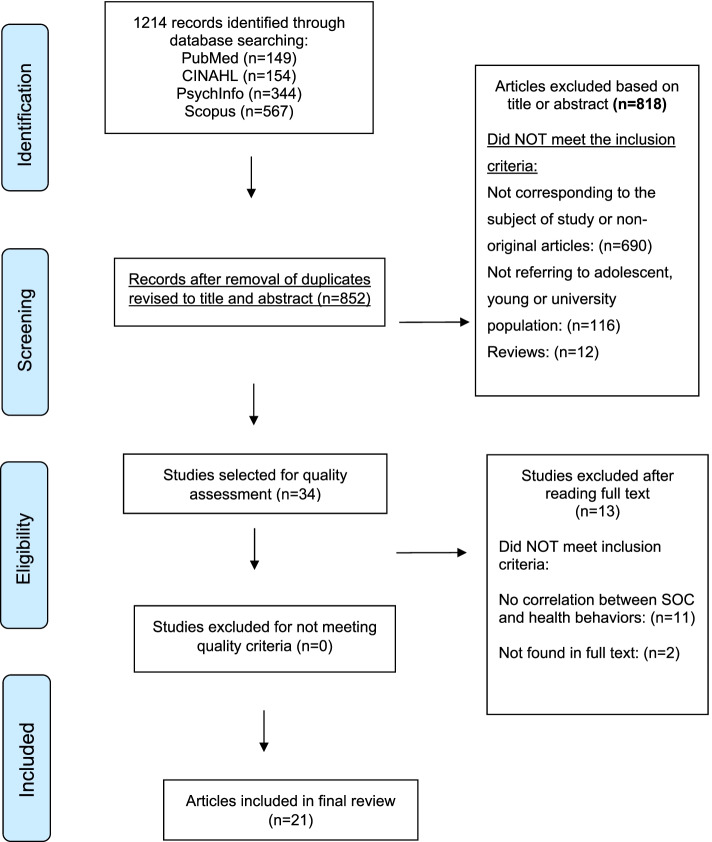
PRISMA flow diagram of the literature review process

Our search strategies allowed us to identify 1214 studies. After removing duplicates and excluding studies that did not meet the inclusion criteria, 21 studies were included [28–48]. Figure 1 shows this process in a PRISMA flow diagram.

With regard to the data, 16 investigations came from adolescents and young adults between 12 and 30 years old [28–30, 33–35, 37–42, 44, 46–48], with a total of 18.454 participants, with an overall average age of 16 years old. Other studies had the participation of 3.529 university students [31, 32, 36, 43, 45]. The characteristics of the included studies in the review are shown in Table 2.

**Table 2:** Characteristics of the studies included in the review

First Author	Objective	N, Age Population	Study design	Dependent Variable	Sampling	Result	Quality
Ayo-Yusuf et al., South Africa, 2009 [28]	To determine the association between adolescents’ sense of coherence and their tooth-brushing behavior.	578 12-19 Young	Longitudinal	Oral health behaviors	Probabilistic	Youth with strong SOC are significantly more likely to brush their teeth twice a day.	High
Ayo-Yusuf et al., South Africa, 2013 [29]	To determine the association between the SOC of nonsmokers and their commitment to remain smoke-free, regardless of the presence of smoking in the home.	1767 13-15 Non-smoking rural youth	Longitudinal	Tobacco use	Probabilistic	Youth with a strong SOC are more likely to remain smoke-free.	High
Bronikowski et al., Poland, 2017 [30]	To investigate the associations of SOC, physical activity, and the role of gender and age in young adolescents.	1296 13-16 Adolescents	Descriptive Cross-sectional	Practice of physical activity	Probabilistic	Adolescents with a strong SOC are positively related to the level of moderate to vigorous physical activity.	High
Chu et al., China, 2016 [31]	Identify factors associated with SOC with emphasis on the impact of perceived stress on the development of SOC.	1853 University students	Descriptive Cross-sectional	Practice of physical activity	Probabilistic	Young adults with a strong SOC practice physical activity ≥3 times per week.	High
DeBruyn et al., United States, 2002 [32]	To address the role that SOC plays in the perception of stress, binge drinking, and convictions to determine the perception of binge drinking norms on campus.	189 University students	Descriptive Cross-sectional	Alcohol use	Probabilistic	Youth with weak SOCs reported binge drinking in the 2 weeks prior compared to those with strong SOCs.	High
Do Carmo et al., Brazil, 2001 [33]	To investigate the relationship between SOC and oral health.	664 15 Adolescents	Descriptive Cross-sectional	Oral health behaviors	Probabilistic	Adolescents with a strong SOC are more likely to visit the dentist for checkups and checkups.	High
Dorri et al., Iran, 2010 [34]	To evaluate the association between SOC and tooth brushing behaviors.	911 11-16 Adolescents	Descriptive Cross-sectional	Oral health behaviors	Probabilistic	Adolescents with a stronger SOC were significantly associated with higher tooth brushing frequencies.	High
El-Shahawy et al., United States, 2015 [35]	To examine the association between SOC, smoking myths and smoking expectancy for the next year and smoking in the past 30 days.	1090 13-19 Young	Longitudinal	Tobacco use	Probabilistic	At baseline, SOC correlated with cigarette consumption and smoking expectancy for the next year, but did not predict changes at follow-up.	Medium
Gajdosova et al., Slovakia, 2009 [36]	To analyze the effect of Eysenck’s personality dimensions, self-esteem and SOC on the probability of being a smoker.	830 University students	Descriptive Cross-sectional	Tobacco use	Non-probabilistic	Male smokers reported higher comprehensibility and meaningfulness, but lower manageability compared to nonsmokers. Girls had lower scores on three SOC dimensions if they were smokers.	Medium
Geada, Portugal, 1994 [37]	To discover the extent to which perceptions of family environment and parental separation influence the development of SOC and how this structure copes with discrimination against illicit drug use behaviors.	471 15-18 Adolescents	Descriptive Cross-sectional	Consumption of illegal substances (cannabis, hashish, heroin, and cocaine)	Non-probabilistic	Adolescents with significantly stronger SOC were non-users of illegal substances compared to users.	Medium
Glanz et al., United States, 2005 [38]	To examine the relationship between ethnicity, SOC and tobacco use.	3438 Average of 12 Adolescents	Descriptive Cross-sectional	Tobacco use	Probabilistic	Adolescents with a strong SOC showed less smoking behavior both ever and during the past 30 days.	High
Grevenstein et al., Germany, 2016 [39]	To examine the incremental validity of SOC on factors for long-term predictors of substance use (cannabis), alcohol, tobacco.	318 14 – 24 Young	Longitudinal	Consumption of illegal substances (cannabis) Alcohol use Tobacco use	Probabilistic	At age of 15 years, having a weak SOC was associated with tobacco use; however, no association was found with alcohol and cannabis use. Having a strong SOC was associated with a reduction in smoking habits at 15 years old and at 24 years old.	High
Kuuppelomäki et al., Finland, 2003 [40]	To measure the strength of SOC and monitor its development for 3 years. In addition, to study the association of smoking, alcohol consumption, and physical exercise with SOC.	284 18-24 Young	Longitudinal	Alcohol use Tobacco use Practice of physical activity	Probabilistic	Strong SOC at baseline was associated with intense physical exercise (more than three times per week). SOC was not associated with smoking and drinking. At the follow-up stage (3 years from baseline) SOC was not associated with smoking, alcohol and physical activity.	Low
Myrin et al., Sweden, 2006 [41]	Investigate the relationship between health behavior and SOC.	383 14-15 Adolescents	Descriptive Cross-sectional	Eating habits Tobacco use Alcohol use Rest periods Consumption of illegal substances (cannabis)	Probabilistic	Girls with a weak SOC were associated with several health-damaging behaviors, with statistical significance in skipping breakfast, skipping dinner, not having sports classes, alcohol consumption and going to bed after 11 pm.	High
Tilles-Tirkkonen et al., Finland, 2015 [46]	To investigate the determinants of regular consumption of a balanced school lunch, with special reference to the role of SOC.	825 10 – 17 Adolescents	Descriptive Cross-sectional	Eating Habits	Non-probabilistic	Adolescents with a strong SOC were associated with a regular intake of a nutritionally balanced school lunch.	Medium
Ullrich-Kleinmann et al., Germany, 2008 [47]	To describe the patterns and course of psychoactive use and to analyze the relevance of risk perceptions and SOC as protective factors.	318 13-16 Adolescents	Longitudinal	Alcohol use Consumption of illegal substances (cannabis)	Probabilistic	Adolescents with stronger SOC were significantly associated with lower alcohol and cannabis use.	High
Ustinavičienė et al., Lithuania, 2018 [48]	To evaluate the relationship between time spent playing computer games and SOC.	1806 13-18 Adolescents	Descriptive Cross-sectional	Time spent in games in the computer	Probabilistic	It was found that in adolescents aged 13 to 15 years old a weak SOC was associated with higher frequency of playing computer action games for 5 or more hours per day.	High

**Fig. 2:**
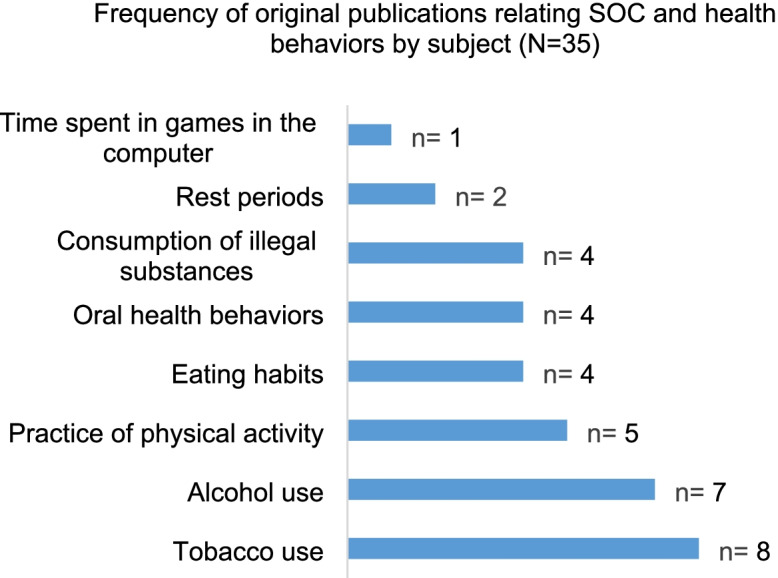
Frequency of original publications relating SOC and health behaviors according to subject matter.^1 1^The total number of dependent variables included in the graph differs from the total number of articles included in the study because some articles related SOC to more than one dependent variable

We identify the relationship of SOC with 8 health behaviors, represented in Fig. 2 between the years 1994 and 2018.

In this review, 14 of the 21 studies included, 66.66% are descriptive cross-sectional correlational designs, which study the relationship between SOC and one or more health behaviors (oral health behaviors, alcohol consumption, tobacco consumption, consumption of illegal substances, physical activity, eating habits, rest periods, time spent in games in the computer). Regarding the type of sampling, two thirds of the articles used probability sampling (66.66%). The studies were carried out in a wide range of countries, including the United States [32, 35, 38], South Africa [28, 29], Sweden [41, 42], Germany [39, 47], Finland [40, 46], Turkey [44, 45], Poland [30], Iran [34], China [31], Brazil [33], Slovakia [36], Japan [43], Lithuania [48], and Portugal [37].

### Assessment of the methodological quality of the included studies

Among the studies with a cross-sectional design, 8 of them [30–34, 38, 41, 47], have a high methodological quality and 6 [36, 37, 42, 44–46] a medium quality. Among those with a longitudinal design, 4 of them [28, 29, 39, 47] presented a high methodological quality, one with medium quality [35] and one with low quality [40], by failing to meet two of the quality criteria (confounding factor and follow-up rate). A cohort design study [43] presented a medium quality (Table 2).

With respect to the measurement instruments, the most widely used version was the short version SOC-13 [30, 33–36, 39, 41, 42, 44–48], six investigations used SOC-29 [28, 32, 37, 38, 40, 43]. In addition, two additional instruments will be used, a 6-item scale [29] and another with 9 items [31]; The instruments used to measure the dependent variables are shown in Table 3.

**Table 3:** Measuring instruments used in the selected articles

Measurement	Name
**SOC**	Sense of Coherence Scale (29 items) [28, 32, 37, 38, 40, 43]
	Sense of Coherence Scale (13 ítems) [30, 33–36, 39, 41, 42, 44–48]
	Six-item adapted Antonovsky SOC scale [29]
	Leipzig Short Scale (SOC-L9) [31]
**Behaviors in health**	**Name**
**Consumption of illegal substances**	Questionnaire ECIT [37] SUF: substance use frequency measure [39] The Health Profile Scale [41] Questionnaire on type and frequency substance use (HSK) [47]
**Tobacco use**	Self-administered questionnaire (Tobacco Use, Commitment to a smoke-free lifestyle) [29] Self-administered questionnaire (past 30-day use of cigarettes, Next-year smoking expectation) [35] Self-administered questionnaire (many cigarettes they smoked per day) [36] Self-administered questionnaire [38] SUF: substance use frequency measure [39] Self-administered questionnaire (health elements) [40] The Health Profile Scale [41] Self-administered questionnaire (health practices) [45]
**Oral health behaviors**	Self-administered questionnaire (dental treatment attendance pattern, frequency of and motivation for tooth-brushing) [28] Self-administered questionnaire (oral health behaviours (dietary habits, oral hygiene, use of fluoride, and dental attendance) [33] Self-administered questionnaire (frequency of toothbrushing more than three times per day, twice a day, once a day, less frequently tan once a day, not at all) [34] Self-administered questionnaire (health practices) [45]
**Practice of physical activity**	Physical Activity Screening Measure [30] Self-administered questionnaire (Physical activity frequency) [31] Self-administered questionnaire (health elements) [40] Physical Activity Assessment Questionnaire (PAAQ) [44] Self-administered questionnaire (health practices) [45]
**Alcohol use**	The Core Alcohol and Drug Survey [32] SUF: substance use frequency measure [39] Self-administered questionnaire (health elements) [40] The Health Profile Scale [41] Self-administered questionnaire (problem behaviour related to alcohol consumption, the frequency of recurring intoxication and items relating to alcohol consumption that inquire about the frequency and consumption levels of beer, wine and spirits. Alcohol-related problem behaviour) [42] Self-administered questionnaire (health practices) [45] Questionnaire on type and frequency substance use (HSK) [47]
**Eating habits**	The Health Profile Scale [41] Self-administered questionnaire (12 items on Lifestyle) [43] Self-administered questionnaire (health practices) [45] Self-administered questionnaire (eating pattern, and the type and frequency of meals) [46]
**Rest periods**	The Health Profile Scale [41] Self-administered questionnaire (health practices) [45]
**Time spent in games in the computer**	Self-administered questionnaire (habits related to the choice of computer game type and time spent playing computer games) [48]

### Relationships between sense of coherence and tobacco use

The relationship between SOC and tobacco consumption was studied in 8 of the included articles [29, 35, 36, 38-41, 45]. A strong SOC was significantly associated with young non-smokers in Turkey. (*p* = .001) [45]. A longitudinal study of young South Africans found that having a strong SOC was significantly more likely to follow a smoke-free lifestyle (*p* = .04) and that the SOC predicted a significantly lower risk of smoking, both ever and during the past 30 days (*p* = .02) [29]. Furthermore, an investigation with American adolescents demonstrated that the three subscales of the SOC are inversely related to smoking (manageability (β = − .45, *p* < .0001), significance (β = − .39, *p* < .0001) and comprehensibility (β = − .28, *p* < .0001)) [38]. However, the longitudinal study conducted in Finland found no association between SOC and smoking risk [40]. A study with university students found no significant association in any of the three dimensions of the SOC in relation to smoking in boys, but in the relationship between the significance subscale of the SOC and smoking there was significant association in non-smoking girls compared to smokers [36].

### Relationships between sense of coherence and alcohol use

The relationship between SOC and alcohol use was investigated in 7 studies [32, 39-42, 45, 47]. A longitudinal study of young Germans found an association between a strong SOC and lower alcohol consumption (*p* = .007) [47]. In American university students [32] association of weak SOC with heavy alcohol consumption was found, compared to those with strong SOC (Chi-square = 21.21, *p* < .05). The longitudinal study conducted in Finland found no relationship between SOC and alcohol use [40]. The research by Nilsson et al. [42] indicates that there is a trend towards an increased likelihood of intoxication, especially among girls with medium and weak SOC (*p* = .001).

### Relationships between sense of coherence and physical activity practice

The relationship between SOC and physical activity was addressed in 5 of the included articles [30, 31, 40, 44, 45]. Positive associations were found between a strong SOC and a higher level of physical activity (B = .024; *p* = .001) in young Poles [30]; Chinese (*p* < .01) [31] and Finns (*p* = .003) [40]. A discrete positive correlation was also found in young Turkishs (*p* < .05; R = .13) [44].

### Relationship between sense of coherence and eating habits

Four of the included articles [41, 43, 45, 46] refer to the relationship between SOC and eating habits. Students with a strong SOC reported consuming sugar less frequently between meals (odds ratio (OR): 0.67; 95% confidence interval (CI): 0.44–0.99) [45]. SOC was also positively associated with the consumption of balanced lunches (*p* = .004) and a regular intake of nutritionally balanced lunch (*p* = .006) in young Finns [46]. In a cohort study in medical students [43] at baseline, stronger SOC scores were found among students who ate breakfast compared to those who skipped breakfast (*p* = .026), but in the second year of study, only the comprehensibility component of the SOC was significantly higher among breakfast eaters compared to breakfast skippers. In Swedish adolescents, girls showed a significantly lower mean SOC value related to skipping breakfast (*p* < 0.05) and not having dinner (*p* < .001) compared to boys [41].

### Relationship between sense of coherence and oral health behaviors

Four articles [28, 33, 34, 45] found that a strong SOC was related to higher frequency of tooth brushing, being in a sample of Turkish university students (*p* = .008) [45], also in young adults in Iran (*p* = .01) [34], and in young adults from South Africa (*p* = .04) [28]. Furthermore, in Brazilian adolescents, a strong SOC was found to be associated with a higher probability of visiting the dentist preventively (OR: 0.83; 95% CI: 0.71–0.98) [33].

### Relationship between sense of coherence and consumption of illegal substances

The relationship of SOC with the use of illegal substances was investigated in 4 articles [37, 39, 41, 47]. A longitudinal study of German students found a lower risk of cannabis use in students with a strong SOC (*p* = .012 U = 673) [47]. Another longitudinal study of German youths found no association between SOC and cannabis use [39]. A Portuguese study found that young non-users of cannabis, hashish, heroin and cocaine had a stronger SOC than users on all three subscales (comprehensibility *p* = .005; manageability *p* = .04 and significance *p* = .0003) [37].

### Relationship between sense of coherence and rest periods

Two articles investigated the relationship of SOC with personal rest periods [41, 45]. In Swedish adolescents, boys with weaker SOC went to bed after 11 pm (*p* < .05) [41]. In Turkish university students, no positive association was found between SOC and resting more than 7 h a day (*p* = .792) [45].

### Relationship between sense of coherence and time spent in games in the computer

A study developed in Lithuania [48] identified that adolescents with a weak or moderate SOC were more likely to play action or combat games for 5 or more hours a day compared to those with a strong SOC (boys *p* = .004; girls *p* = .027).

## Discussion

In the present study we have analyzed the available scientific production that relates the Sense of Coherence with health behaviors in the adolescent and young population. This review shows that 8 health behaviors were investigated, including both preventive behaviors (physical activity, eating habits, rest periods or oral health behaviors) and risk behaviors (consumption of illegal substances, alcohol use, tobacco use and time spent in games in the computer). The relationship between SOC and health behaviors is not much researched so far, only 21 original articles met the inclusion criteria of this review.

The SOC-13 questionnaire is the most widely used in the research reviewed; although the reason for its choice is not made explicit in the studies with respect to other alternatives such as SOC-29. In addition, we have found a large number and heterogeneity of instruments to measure health behaviors, making it difficult to combine and compare the results, so it has not been possible to use statistical methods such as meta-analysis. The same difficulty related to the heterogeneity of the measurement instruments on health behaviors was found by Aceijas et al. in their review on health-related lifestyles in students [49].

Our findings identified studies on the relationship of SOC with four risk behaviors; the most studied refers to tobacco use and the results indicate that a strong SOC is positively associated with lower smoking frequency, but one study does not find this association [40]. In relation to alcohol consumption, a positive association was found between a strong SOC and low consumption of alcoholic beverages; in three studies this relationship was not found [39, 40, 45]. Having a strong SOC is associated with low use of cannabis, hashish, heroin, and cocaine. In addition, evidence was found that adolescents with strong SOC are less likely to play computer games for more than 5 h a day. Regarding the relationship between time spent playing computer games and SOC, it is striking that in our research we found only one study that investigated this relationship, yet there is now sufficient scientific evidence to suggest that excessive time spent playing computer games could be a health problem for individuals and a tendency towards addiction [50–52].

The interpretation of the fact that some studies did not find an association between SOC and risk behaviors may be diverse; among other factors, there could be other unstudied variables involved in this relationship. Antonovsky himself [11] proposed that SOC tends to stabilize in early adulthood and that conditions present before this phase (adolescence or youth), such as economic conditions, social relationships, life experiences and culture influence the development of SOC, which could intervene in health behaviors. Research conducted in adults augments the evidence of most of the studies included in our review, demonstrating the association between strong SOC and low substance use, tobacco [53], alcohol and illegal drugs [54].

Regarding the relationship between SOC and preventive health behaviors in adolescents and young adults, we have found that the practice of physical activity has been the most studied, followed by oral health behaviors, eating habits and rest. Associations were significant for most behaviors, with the exception of one investigation of physical activity and one investigation of rest period [45]. The data provided by our study, taken as a whole, support Antonovsky’s salutogenic theory, in the sense that having a strong level of SOC may favor appropriate and positive choices regarding health behaviors. Individuals with a strong SOC may be better prepared to respond to internal and external influences during the early stages of life: adolescence and the transition to adulthood. Strong SOC is associated with healthier behavioral choices, while a weak SOC is associated with unhealthy behaviors. In his research, Wainwright [55] concludes that individual differences in SOC are associated with healthier behavioral choices in health regardless of social class and education. Our results are in line with those of other studies such as Edbom et al. [56] that support the idea that a strong SOC is a protective factor favoring positive health behaviors and a protective factor against health risk behaviors. The findings of our review were similar to other studies on adults where it was found that a strong SOC is related to regular visits to the dentist for prevention [57]; in Japanese workers with a weak SOC, a relationship was found with health-damaging behaviors, including lack of physical exercise [58]; a strong SOC was also found to be a predictor of healthier food choices [59].

The ages of the groups included in our study were similar; also the cultural diversity of the groups (13 countries on 5 continents) prefigure a global vision of adolescents and young adults. Future research should consider how sociocultural and educational aspects influence the relationship between SOC and health behaviors.

A minority of studies have analyzed the difference in SOC scores with respect to gender, finding that, in most of them, girls have a stronger SOC than boys, presenting less frequently risky behaviors (time spent in games in the computer [48], consumption of alcohol [42] and tobacco [36]) and more preventive health behaviors (rest periods and eating habits [41]). For future research, we propose the importance of investigating the relationship between gender, the process of SOC development and health behaviors.

Our study provides expanded results over the previous review of Länsimies [20] which describes the relationship between SOC and adolescent health; part of this study analyzes the relationship between SOC and health behaviors, identifying three behaviors investigated so far: alcohol consumption, tobacco consumption and drug use in adolescents. Our work extends the list of health-related behaviors to eight; nevertheless, we believe that the study of the relationship between SOC and health behaviors is insufficient, as only 21 original studies have been identified.

Our bibliographic search strategy has been carried out without restrictions of any kind and has been discussed and agreed upon by the entire research team. It is noteworthy that behaviors related to sexuality, affectivity or safety of adolescents and young adults have not been studied to date.

This systematic review has some methodological limitations; the first of which relates to the great heterogeneity and variability in the instruments used to measure health behaviours. A second issue is related to the methodological quality of the selected studies, which may lead to selection bias. In addition, most of the studies are cross-sectional descriptive studies, so that no causal relationship can be inferred. It would be advisable to develop longitudinal studies in future research. On the other hand, a consensus on the use of questionnaires for the study of health behaviors would be desirable, which would facilitate the comparability of the results and the use of advanced statistical techniques (meta-analysis).

## Conclusions

Our results support the strong relationship of SOC with health behaviors; on the one hand, as a protective factor against risk behaviors and on the other hand, because of its positive association with preventive health behaviors in youth and adolescents.

This study has shown that the use of SOC as an independent variable contributed to a deeper understanding of the influence of SOC on the health behaviours of young people and adolescents. A strong SOC was associated with less smoking, less alcohol consumption, higher levels of physical activity, better eating habits, less use of illegal substances, more frequent tooth brushing, more rest periods and less time spent playing computer games.

Our review shows that research relating SOC and health-related behaviors in adolescents and young people is so far underdeveloped. We underline the importance of further research on this population, as it constitutes an important population group from a public health point of view, mainly because their current health habits and behaviours will have a decisive impact on their lifestyles and health status in the future. In addition, we emphasise the absence of studies on the relationship of SOC with sexual and reproductive behaviors, safety or violence.

For future research, more longitudinal studies are needed to improve the causal relationships between SOC and health behaviours. More attention should be paid to the instruments used, avoiding, whenever possible, the use of self-reports that imply a limitation of data collection.

The data provided by our research support the salutogenic theory by solidly establishing that a strong SOC is related to healthier and lower risk behaviors. The study of gender differences is still a minority in the research analyzed.

## Data Availability

All data generated or analysed during this study are included in this published article in both tables and references.
